# A Low-Noise Micromachined Accelerometer with Reconfigurable Electrodes for Resonance Suppression

**DOI:** 10.3390/mi14061188

**Published:** 2023-06-02

**Authors:** Zayed Ahmed, Charles Duruaku, Fatemeh Edalatfar, Mehrdad Moallem, Behraad Bahreyni

**Affiliations:** School of Mechatronic Systems Engineering, Simon Fraser University, Burnaby, BC V5A 1S6, Canada

**Keywords:** high-performance accelerometers, resonance suppression, vacuum packaged accelerometer, negative derivative controller

## Abstract

We present a high-performance capacitive accelerometer with a sub-µg noise limit and 1.2 kHz bandwidth for particle acceleration detection applications. The low noise of the accelerometer is achieved through a combination of device design optimization and operation under vacuum to reduce the effects of air damping. Operation under vacuum, however, causes amplification of signals around the resonance region, potentially resulting in incapacitating it through saturation of interface electronics or nonlinearities and even damage. The device has thus been designed with two sets of electrodes for high and low electrostatic coupling efficiency. During normal operation, the open-loop device utilizes its high-sensitivity electrodes to provide the best resolution. When a strong signal near resonance is detected, the electrodes with low sensitivity are used for signal monitoring, while the high-sensitivity electrodes are used to apply feedback signals efficiently. A closed-loop electrostatic feedback control architecture is designed to counteract the large displacements of the proof mass near resonance frequency. Therefore, the ability to reconfigure electrodes lets the device be used in high-sensitivity or high-resiliency modes. Several experiments were conducted with DC and AC excitation at different frequencies to verify the effectiveness of the control strategy. The results showed a ten-fold reduction of displacement at resonance in the closed-loop arrangement compared to the open-loop system with a quality factor of 120.

## 1. Introduction

The current market demand in a wide range of industries, including automotive, aerospace, medical, consumer electronics, and space exploration, is fueling the development of accelerometers with improved performance metrics such as resolution or bandwidth. The small size, batch production, low cost, and high performance of micromachined accelerometers drive their market expansion. Even though micromachined accelerometers dominate the low-end commercial industry, they have struggled to expand to high-performance markets with significantly higher profit margins. For instance, the vector sensing nature of accelerometers allows them to be used in high-reliability and low noise-based applications such as underwater particle acceleration [1], gravity wave detection, and geo-acoustics seismic wave detection [2]. Lately, research is being conducted to push the boundaries of accelerometer performance metrics such as noise floor, resolution, and bandwidth to utilize them in more demanding applications.

Capacitive accelerometers have shown remarkable linearity, robustness, and sensitivity, making them highly desirable for many applications [3]. However, their structure poses a challenge in developing a low-noise device stemming from the small sensing gap between the electrodes needed for high sensitivity. As a result, the noise floor of the system is affected by both electrical and mechanical noise, which could limit the sensitivity and accuracy of the device. Numerous studies have been conducted to reduce the total noise floor of the system, which includes electrical and mechanical noise. There have already been well-established works presented in the literature for low-noise electronics, while mechanical noise floor improvement remains unexplored. Models indicate that mechanical noise floor can be reduced by either designing a large-mass accelerometer or operating the sensor under a vacuum to reduce damping. A significant challenge with large mass devices is the low yield factor during fabrication, even when the dimensions are within the fabrication limits. Another method of lowering the noise floor involves maintaining a low damping factor in the system, which means increasing the device’s quality factor (Q). High-Q devices amplify signals at resonance, which refers to the frequency at which the device exhibits maximum response or sensitivity. This resonance allows the device to enhance the amplitude of incoming signals efficiently. When a high-Q device operates at resonance, it experiences sustained oscillations (depending on Q) characterized by a prolonged ringing response at the output. The amplified oscillation within the device results in Q times having larger displacement than the static displacement. The “Q” value, or quality factor, represents the efficiency and selectivity of the device’s resonance. A higher Q value indicates a narrower resonance bandwidth and a more pronounced amplification effect. However, along with the advantages of amplification, high-Q devices also present certain vulnerabilities.

One such vulnerability is electrode snapping, when two electrodes within the device experience an increased attraction force during amplified oscillations. This heightened attraction can lead to the electrodes touching each other, causing a short circuit and disrupting the device’s operation and even permanent damage.

Another consequence of the substantial signal amplification achieved by high-Q devices is amplifier saturation. Amplifier saturation occurs when the amplification capabilities of the device reach their maximum limit. In this state, the amplifier becomes overloaded, unable to increase the signal amplitude further. As a result, the device’s output signal becomes distorted, compromising its ability to process and transmit information accurately.

To ensure the reliable operation and longevity of high-Q devices, it is important to address the risks associated with electrode snapping and amplifier saturation, considering measures that mitigate these issues while maintaining the desired low-noise response that is achieved with a high-Q sensor.

Researchers have tried different approaches for suppressing the resonance of the proof mass, including passive and active methods of introducing artificial damping. One of the most effective methods of resonance suppression is shifting the system’s natural frequency by altering the lengths of the structural elements. However, this would require a nonlinear spring design and its required controller [4]. Yoon et al. reported a shock impact reduction technique by introducing nonlinear spring and soft coatings on hard silicon surfaces [5]. Diveyev et al. reported on optimizing a dynamic vibration absorber for a selective resonance mode of a MEMS device [6]. Bottenfiled et al. designed and implemented a microscale vibration isolator for attenuating high-frequency excitation signals beyond a cutoff frequency to artificially reduce the system’s effective quality factor [7].

Another approach presented in the literature is based on pumping energy actively into the microsystem to rebalance the inertia force generated by the external acceleration. This approach uses a pair of capacitive microactuators to generate a linear feedback rebalancing force and establish a closed-loop system. Researchers have used either digital [8] or analog interfaces for closed-loop control system implementations. Yazdi and Najafi [9] implemented a second-order sigma-delta (∑Δ) interface for a capacitive accelerometer with Q of 16. Other researchers, including Almut [10] and Xu [11] demonstrated a higher-order stable ∑Δ interface for moderately high Q capacitive accelerometers. Although the ∑Δ interface offers a good resolution and low noise floor for low-Q accelerometers, the design becomes complicated and can lead to instability for high-Q systems [8].

Continuous-time implementations of closed-loop systems have also been reported in the literature to attain linearity, suppress large displacements, or manage the sensor to work beyond the pull-in region. The most extensively used compensators for establishing a closed-loop continuous interface are PI [12], PD [13,14,15], and sliding mode controllers. Another research reported by Sarraf [16] implemented a D controller for handling a Q of 2000. A recent work by Lavinia [17] utilized a PID and ∑Δ for implementing a stable closed-loop system for an accelerometer with a Q of 200. Table 1 summarizes the work performed by other researchers for implementing the controller.

To date, most reported under-damped closed-loop accelerometers have utilized relatively small proof masses. As such, countering the inertial forces has been possible using actuation voltages that are easily attainable (typically less than 10 V). However, the required voltages to stabilize accelerometers with large proof masses can require large actuation voltages (e.g., >200 V for the device presented in this paper). Large actuation voltages are particularly needed if the accelerometer is exposed to strong random or intentional (i.e., jamming) signals near the device’s resonant frequency. In this paper, we demonstrate a reconfigurable system architecture where a compensator is proposed for suppressing the large displacement of a high-Q, large-mass accelerometer. The compensator reported in this work is frequency-dependent and only works in the desired operating region to avoid introducing additional noise.

The organization of the paper is as follows. Section 2 discusses the basic principles of micromachined seismic accelerometers and the fabrication method for the device used in this research. Section 3 elaborates on the open-loop characterization of the device. Section 4 discusses closed-loop system design. Finally, the paper concludes with implementation and results.

## 2. Micromachined Seismic Accelerometers

**Figure 1:**
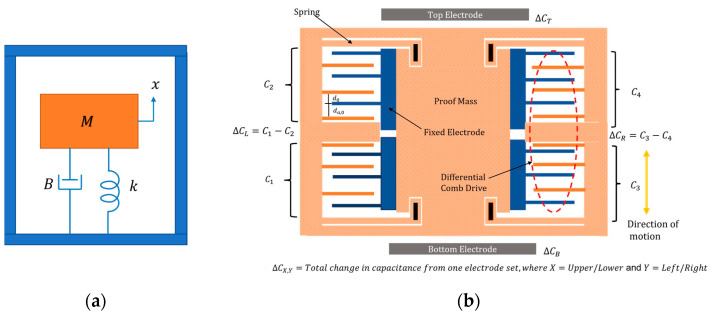
(**a**) Generic schematic of a seismic accelerometer, and (**b**) the schematic of ISLX showing the high-sensitivity electrodes C1 to C4 and low-sensitivity electrodes CT and CB.

Micromachined accelerometers are electromechanical devices that convert external acceleration exerted on the device to a measurable electrical quantity. For most devices, displacements of a suspended proof mass relative to its frame are measured to estimate the input acceleration. The mechanical structure of an accelerometer can be modeled as a 2nd order system with a lumped spring (k), mass (M), and damper (B) system, as shown in Figure 1a. Balancing the system mechanical response to the exerted force, the equation of motion can be written as

(1) $$M\frac{d^{2}x}{dt^{2}}+B\frac{dx}{dt}+kx=Ma_{external}$$

where B is the damping coefficient.

The quality factor is a dimensionless parameter that describes the damping in the system. A higher quality factor indicates that the system has better resolution but also increases settling time. Taking the Laplace transform of Equation (1), and noting that natural frequency and quality factor of the system are found from $\omega_{0}=\sqrt{k/M}$ $\mathrm{and}\;Q=\frac{\omega_{0}M}{b}$, respectively, the system transfer function from the input acceleration to output displacement can be written as

(2) $$\frac{X\left(s\right)}{a\left(s\right)}=\frac{1}{s^{2}+\frac{\omega_{0}}{Q}s+\omega_{0}^{2}}=\{\begin{array}{c} \frac{M}{k}=\frac{1}{\omega_{0}^{2}}\;\;\;\;\;\;\;\;\;\;\;\omega \prime \prime \omega_{0}\\ Q\frac{M}{k}\;\;\;\;\;\;\;\;\;\;\;\;\;\;\;\;\;\;\;\;\;\omega =\omega_{0}\;\;\;\\ \frac{1}{\omega^{2}}\;\;\;\;\;\;\;\;\;\;\;\;\;\;\;\;\;\;\;\;\;\;\;\omega \prime \prime \omega_{0}\;\;\; \end{array}$$

As is evident from Equation (2), the bandwidth of the accelerometer is mainly limited to its first natural frequency, $\omega_{0}$. At frequencies lower than the natural frequency, the accelerometer’s sensitivity to the input is constant. At resonance, the accelerometer’s response is amplified by a factor of Q compared to its static value. This amplification in the system response can create mechanical and electrical failure.

The total noise equivalent acceleration depends on mechanical noise equivalent acceleration (MNEA) and electrical noise equivalent acceleration (ENEA) [20] and can be written as

(3) $$TNEA=\sqrt{ENEA^{2}+TNEA^{2}}$$

ENEA is the electronic noise and depends on the interface circuit that is used to convert the signals from the mechanical to the electrical domain. Considering that one often has more flexibility in designing the interface electronics from various integrated or discrete components and topologies, the limit of noise performance is often set by MNEA, which is the mechanical noise and depends on the mass (M), bandwidth ($\omega_{0})$, quality factor (Q), and temperature of the system (T):

(4) $$MNEA=\sqrt{\frac{4K_{B}T\omega_{0}}{MQ}}\;\;\;\;\;\;\;\;\;\;\;\;\;\left[\;\frac{{\mathrm{ms}}^{-2}}{\sqrt{\mathrm{Hz}}}\right]$$

where $K_{B}$ is Boltzmann’s constant. As can be seen, the only practical possibility to reduce the noise of seismic accelerometers with a given operational bandwidth is to increase either their mass or quality factor (i.e., lower damping). However, increasing mass for micromachined accelerometers is challenging due to the limitations on lateral dimensions of the devices and the small thicknesses of structural layers in microfabrication processes. These limitations have been behind the trend toward increasing the quality factors of these devices by operating them in a vacuum.

### 2.1. The ISL Accelerometer, ISLX

A high-performance, single-axis accelerometer, herein referred to as the *ISL accelerometer (ISLX)*, designed and fabricated by our team at the Intelligent Sensing Laboratory (ISL), is used for this research. This device was designed as a vector particle acceleration sensor to detect acoustic waves. The design objectives were therefore tuned toward optimum sensitivity and noise performance over the operational bandwidth of the device. The device offers a sub-$\mu g/\sqrt{\mathrm{Hz}}$ noise level (depending on vacuum level) with a sensitivity of 1 V/g over the frequency range of 50 Hz to 1.2 kHz.

**Figure 2:**
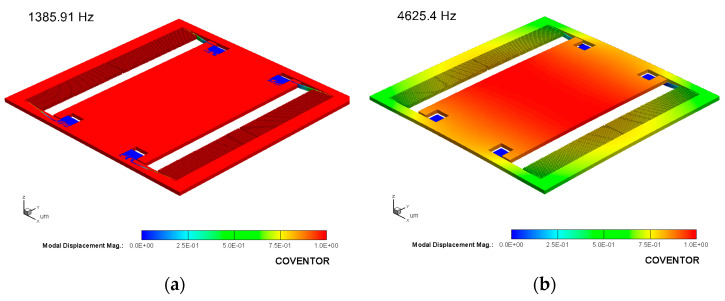
The simulated first (**a**) and second (**b**) mode shapes and frequencies for the ISLX.

The ISLX has two pairs of transverse capacitive comb drives to detect proof-mass displacements and a pair of fixed-plate parallel capacitive elements for testing or actuation purposes. The structure of the accelerometer is shown in Figure 1b, and its dimensions are provided in Table 2. The static and dynamic responses of the device were analyzed using the finite element method in Coventorware. Figure 2 shows the first two mode shapes and frequencies of the accelerometer. The first resonance frequency of the accelerometer is found to be in plane vibration mode with a frequency of 1.3 kHz. By design, the structure’s second and higher resonance modes are at significantly higher frequencies.

For the high-sensitivity comb electrodes, each moving finger connected to the proof mass is placed next to a fixed finger with a gap of $d_{0}$ while the gap to the next fixed finger is $d_{0,a}\prime \prime d_{0}$. When the proof mass moves upward, the gap in the upper sets of electrodes ($C_{2}\;\mathrm{and}\;C_{4})$ widens, reducing their capacitances, while the capacitances of lower electrodes ($C_{1}\;\mathrm{and}\;C_{3})$ increase. Capacitance in the upper set of electrodes is:

(5) $$C_{2}=N\frac{\varepsilon_{0}A}{d_{0}-x}+\left(N-1\right)\frac{\varepsilon_{0}A}{d_{0,a}+x}$$

where N is the number of fingers, $d_{o}$, N−1 is the number of capacitors formed by anti-gap, $d_{0,a}$, A is the overlapping area in each finger pair, $\varepsilon_{0}$ is the permittivity of air, and x is the displacement of the proof mass. For the design purpose, the anti-gap is set to be larger than the nominal gap ($d_{0,a}\approx 3.5\;d_{0}$). Hence, the capacitance formed by the anti-gap can be ignored, and the net capacitance change can be written as

(6) $$\Delta C=C_{2}-C_{1}=\frac{N\varepsilon_{0}A}{d_{0}-x}-\frac{N\varepsilon_{0}A}{d_{0}+x}=2N\varepsilon_{0}A\frac{x}{d_{0}^{2}-x^{2}}\approx 2\frac{N\varepsilon_{0}A}{d_{0}^{2}}x$$

The low-sensitivity electrodes form two parallel-plate capacitors above and below the proof mass, as shown in Figure 1.

### 2.2. Fabrication Process

**Figure 3:**
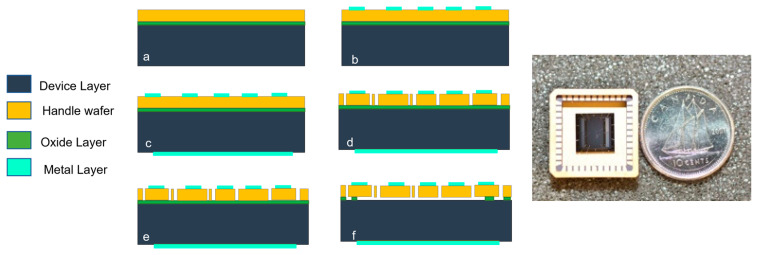
ISLX fabrication process (**left**): (**a**) starting SOI wafer with 100 μm device layer, a 5μm BOX, and a 500 μm handle wafer; (**b**) deposition and patterning of metal pads and traces; (**c**) backside metal deposition; (**d**) deposition and patterning of hard-mask and DRIE of the device layer; (**e**) diced chip; (**f**) die-level device release in vapor HF; and the packaged ISLX (**right**).

The ISLX accelerometer was fabricated from a P-type (100) silicon-on-insulator (SOI) wafer using bulk micromachining techniques developed by Edalatfar et al. [21]. The wafer used for the microfabrication process has a 100 μm thick device layer, a 5 μm buried oxide layer (BOX layer), and a 500 μm handle wafer. Figure 3 illustrates the fabrication processes of the accelerometer. The process requires two lithography masks and a shadow mask. First, the metal contact pads of the device were defined using one of the lithography masks. In the next step, a shadow mask was utilized to deposit chromium and gold using a physical vapor deposition technique on the backside of the wafer as required for packaging. A hard mask is formed by depositing a thin silicon dioxide (SiO_2_) film on top of the device layer using chemical vapor deposition. This SiO_2_ mask layer was patterned using the second lithographic mask by reactive ion etching. Subsequently, the mask layer was used to pattern the device layer through a deep reactive ion etching (DRIE) process. Finally, the wafer was diced, and the structure was released by etching the buried oxide layer and the remaining top oxide layer in vapor hydrofluoric acid. Finally, the devices were packaged in leadless ceramic packages for testing, as shown in Figure 2c. These devices were tested at various ambient pressure levels to determine their sensitivity and frequency response.

## 3. Open-Loop Characterization

**Figure 4:**
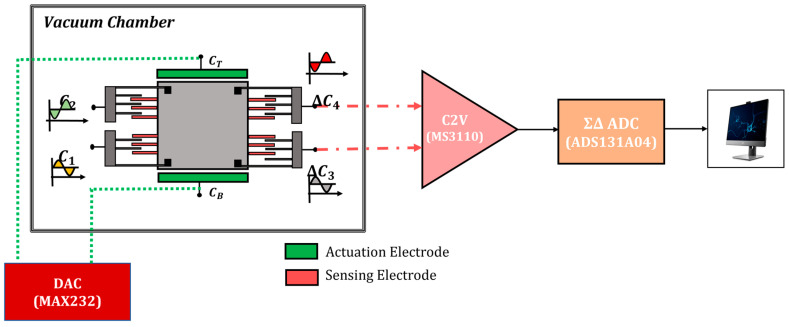
Test setup for open-loop testing of ISLX.

The device was placed inside a vacuum chamber and characterized under different pressures. Figure 4 shows the arrangement for the experimental setup. To evaluate the open-loop characteristics of the sensor, the device was tested with sinusoidal excitation at various frequencies under different pressure levels. The device can be excited mechanically (using a shaker) or electrostatically (by applying a voltage to its top or bottom electrodes). However, mechanical excitation introduces the issue of unwanted resonance peaks from the fixture, circuit boards, or other members of the test setup. Moreover, placing the shaker inside a vacuum chamber is impractical due to size and contamination issues. Hence, the device was electrostatically excited by using an electrode pair on the top and bottom of the proof mass. A digital-to-analog converter (DAC) generating biased sinusoidal signals swept from 50 to 1800 Hz for frequency response estimation. The applied voltage to the excitation electrodes can be converted to equivalent acceleration through:

(7) $$a_{external}=\frac{2\varepsilon_{0}A_{a}}{Md_{a}^{2}}V_{Dc}V_{Ac}$$

where $A_{a}$ and $d_{a}$ are the area and gap for the actuation electrodes and $V_{DC}$ is the bias voltage needed to linearize the nonlinear electrostatic force on the proof mass.

### 3.1. Frequency Response Analysis

**Figure 5:**
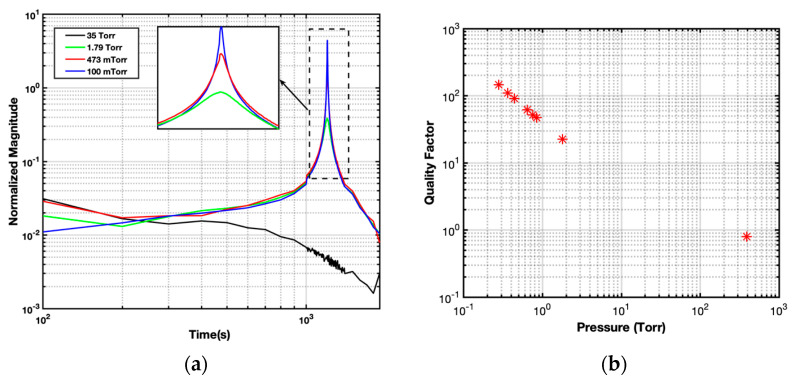
(**a**) The open-loop frequency response of the accelerometer to electrostatic excitation. (**b**) Relationship between pressure and quality factor for the ISLX.

Using this test setup, a DC bias voltage of 200 mV along with a 50 mV (peak-to-peak) differential AC signal were applied to the drive electrodes ($C_{T}$,$\;C_{B}$). The applied electrostatic force is equivalent to a 1 mg input acceleration signal. As seen in Figure 5a, the resonance frequency of the system was found to be around 1220 Hz. Furthermore, as the pressure in the system was reduced from 35 Torr to 100 mTorr, the peak at resonance became sharper and stronger, indicating a transition from an overdamped to an underdamped state. The quality factor of the accelerometer for each pressure level is derived by dividing the resonance frequency by the −3 dB bandwidth from the frequency response curve. Figure 5b demonstrates that the accelerometer’s quality factor is inversely proportional to vacuum pressure.

### 3.2. Sensitivity Measurement

**Figure 6:**
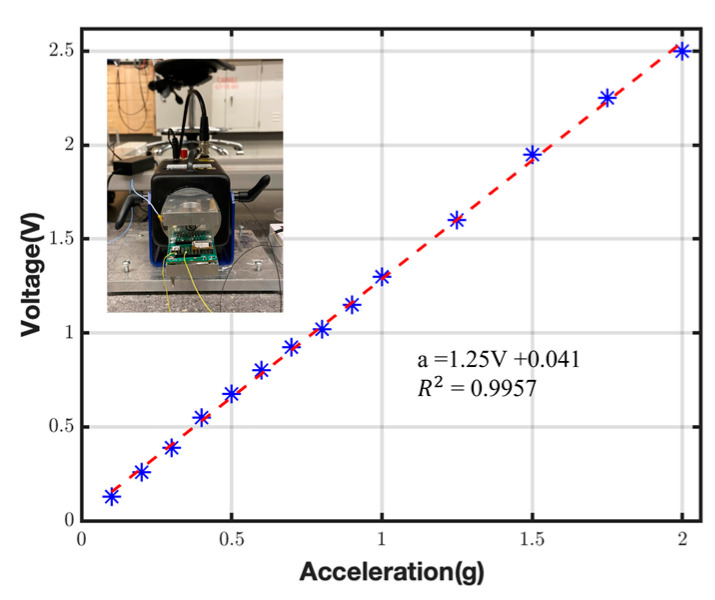
The output response of the accelerometer for various input accelerations at 200 Hz (dashed line is the fitted linear regression and * is the experimental data).

To investigate the output response of the device, the accelerometer was tested under atmospheric pressure with different applied acceleration values ranging from 2 mg to 2 g at 200 Hz. The test was performed by mounting the device on a mechanical shaker (ModalShop K2004E, Cincinnati, OH USA) along its sensing axis. The shaker data acquisition system recorded the output response. A piezoelectric reference accelerometer was used to monitor the shaker’s dynamic properties. Figure 6 shows the output response of the sensor for different acceleration inputs. The overall sensitivity and the nonlinearity of the accelerometer are ~1 V/g and 0.1%, respectively.

## 4. Closed-Loop System Design

**Figure 7:**
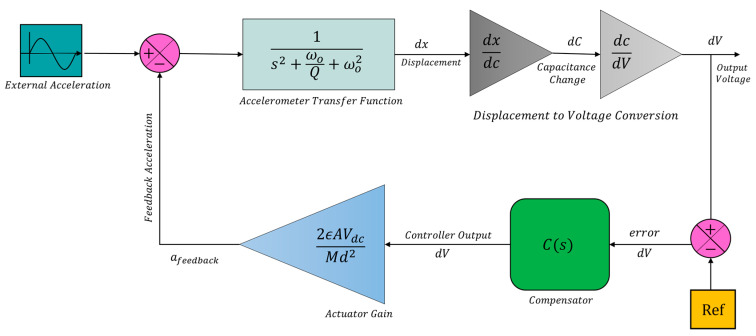
Closed-loop system architecture of ISLX.

The main idea of a closed-loop system is to counterbalance the proof mass displacement by applying an equal force that opposes the external inertial force imparted on the system. From the control system design standpoint, this problem can be regarded as a tracking or regulation problem. In a tracking control problem, the measured acceleration tracks the unknown acceleration applied to the system to maintain the reference point [22]. Alternatively, external acceleration is considered as a disturbance in a regulation-type control problem and is rejected by exerting an electrostatic force in the opposite direction. Regardless of the control problem topology, the goal of a closed-loop control system is to maintain the proof mass at rest despite external acceleration. Figure 7 shows a block diagram of the closed-loop control system where the output voltage representing displacement information is sampled, scaled by a compensator, and fed back to the system through electrostatic actuators.

The ISLX has one pair of fixed electrodes and two pairs of sensing electrodes. In the presence of an external acceleration, the proof mass moves and causes a capacitance variation between sensing electrodes ($C_{1}$, $C_{2},\;C_{3},\;C_{4}$). It is then converted to voltage using capacitance-to-voltage circuitry. This signal then passed through the compensator to produce the counteracting feedback force on the proof mass. The top and bottom electrodes ($C_{B}$ and $C_{T}$) are to be used as actuators. A fixed DC voltage is added to the electrode, which causes the electrostatic actuator to behave linearly and generate a bidirectional force [23]. The net electrostatic force ΔF applied to the proof mass is [24]:

(8) $$\Delta F=\frac{1}{2}\frac{dC}{dx}\left[{\left(V_{DC}+V_{f}\right)}^{2}-{\left(V_{DC}-V_{f}\right)}^{2}\right]=2\frac{dC}{dx}V_{DC}V_{f}$$

where $V_{f}$ is the feedback voltage and $V_{DC}$ is the fixed DC bias voltage, and C is the capacitance of the actuator electrodes.

However, the top and bottom actuators required impractically large voltages to effectively compensate for the movements of the proof mass (on the order of 200 V for a 1 g input). On the other hand, the system’s sensing electrodes comprise a large number of interdigital fingers (69 per comb). Compared to the top and bottom electrodes, the sensing electrodes’ capacitance is 60 times higher due to the large number of fingers and narrower gaps between them. Thus, the force generated by the sensing electrode would be 60 times larger than the designated actuation electrode for the same applied potentials. Therefore, one possibility is to use a pair of sensing electrodes as actuators while keeping the other pair for sensing. However, this would result in a loss of sensitivity, affecting the device’s performance.

**Figure 8:**
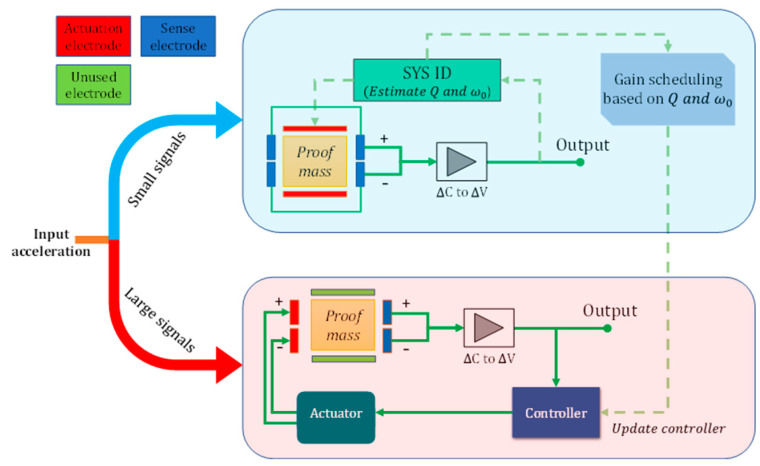
Alternating electrode arrangements for ISLX (dashed lines indicate occasional connections). Under linear operation conditions (i.e., small signals), the device operates in an open loop with maximum sensitivity. A system identification module measures the device response occasionally to update the controller. When strong signals near resonance are detected, the electrodes around the device are rearranged to allow for actuation with reasonable voltages at the expense of reduced sensitivity.

Figure 8 shows the arrangement of electrodes under different operating conditions. The accelerometer normally operates in an open-loop configuration, which lets it use the most sensitive capacitive elements to detect weak signals. A system identification algorithm is run occasionally (from once an hour to once a day) to extract the device’s resonance frequency and quality factor, which are needed for the controller design. This algorithm uses the top and bottom electrodes to apply a swept sine wave to the proof mass while detecting its response using the sense electrodes. Once strong signals near the resonance frequency of the device are detected, the electrode arrangement is changed using on-board electronic switches such that one of the two pairs of sensing electrodes is used for actuation and the remaining pair is used to detect incoming signals. In this case, the magnitude of actuation voltages is on the order of volts to compensate for hundreds of mg-level signals. The feedback loop is closed using the (updated) controller to reduce the quality factor. Once these signals are removed, the system reverts to its high-sensitivity mode of operation (open-loop).

The requirement for a compensator stem from the need to stabilize the system and suppress the large displacements at resonance. The mechanical sensing element at resonance exhibits a Q-fold increase in displacement compared to its low-frequency response. Furthermore, around the resonance frequency, the mechanical element begins to lag in proportion to the input acceleration, resulting in a −90° phase shift. As the quality factor increases, system poles move near the jω axis, resulting in a narrower −3 dB bandwidth with a steeper phase change, making the controller design challenging. One of the fundamental requirements for the controller is to calculate the amount of voltage that must be applied to the electrode to generate an equivalent amount of force that counteracts the external inertial force imposed on the proof mass and compensates for the phase change at resonance.

Moreover, there are additional requirements that must be met in order to design an appropriate controller. First, the controller needs to be frequency dependent. It should only add gain to a specific frequency range, while at other frequencies it should allow the system to work seemingly as an open-loop one. Furthermore, the controller design must account for the uncertainties in the fabrication as well as the variation in the system’s parameters such as quality factor and resonance frequency over time. To accommodate the changes in the system, the controller would either have to be adaptive or robust enough to handle the uncertainties. Finally, another critical consideration when designing the controller is the unmodeled dynamic system’s out-of-band modes. Mechanical systems are continuous systems governed by partial derivative equations. However, for the simplicity of controller design, the higher-order terms of the system are frequently ignored, resulting in a reduced-order model [25].

The appropriate controller should only impact the overall damping of the system around resonance. A derivative controller can provide sufficient damping to the system by increasing the damping factor in the velocity term as shown by the system’s equation of motion, given by

(9) $$M\ddot{x}+\left(B+\beta \right)\dot{x}+kx=Ma_{ext}$$

where $\beta =2\varepsilon_{0}AV_{DC}d^{-2}$ is the electrostatic damping coefficient [23].

The system’s wide bandwidth, however, implies that a derivative controller could magnify noise and may cause instability. Thus, implementing a derivative controller alone is not a feasible option. To address this issue, a new type of controller is used here: a frequency-dependent controller that operates on the desired frequency range of near resonance. Over this range, the controller will amplify the feedback signal and compensate for the phase change. A bandpass filter, along with a derivative controller, is used to form a resonant controller [25]. The controller produces no phase change at resonance so the signal can be fed back as is. The bandpass filter formulation reduces the chances of spillover at high frequencies while limiting low-frequency response degradation [26].

If the $\dot{\eta }\left(t\right)$ is the derivative of the controller output, and x is the displacement of the structure, then the controller can be implemented using a second-order dynamic system as:

(10) $$\ddot{\eta }\left(t\right)+\frac{Q_{c}}{\omega_{c}}\dot{\eta }\left(t\right)+\omega_{c}^{2}\eta \left(t\right)=k\dot{x}\omega_{c}$$

where $Q_{c}$ and $\omega_{c}$ are the quality factor and frequency of the compensator, respectively, and *k* is the controller gain. The transfer function of the controller can be represented as

(11) $$C\left(s\right)=\frac{\eta \left(s\right)}{\;X\left(s\right)}=k\frac{\omega_{c}s}{s^{2}+\frac{\omega_{c}}{Q_{c}}s+\omega_{c}^{2}}$$

**Figure 9:**
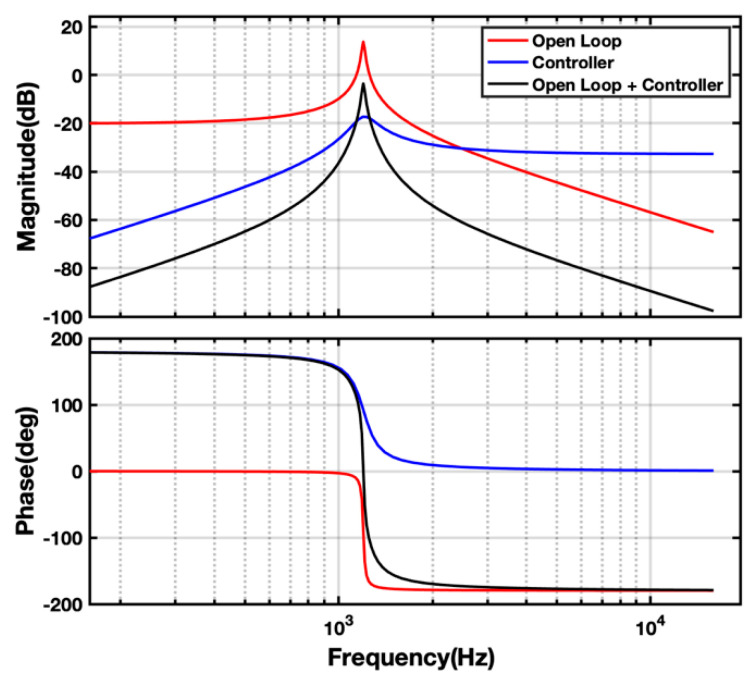
Modeled frequency response of ISLX components.

The controller frequency is set to be the same as the system resonant frequency while $Q_{c}$ determines the bandwidth of the controller. A high $Q_{c}$ yields narrower bandwidth and higher gain at that specific frequency. However, this might also push the system to instability. The optimal value of $Q_{c}$ is thus found empirically and is chosen to be 6 in this work. Figure 9 shows the frequency response of the controller path (open loop + controller). It is evident that with the central frequency set to 1220 Hz and quality factor to 6, the controller path shows +180° phase change at resonance. Hence, the output of the controller can be fed back negatively to the system to generate a cancelling effect. The proposed controller is implemented digitally on a microcontroller (also responsible for system identification and switching of the electrodes as needed) and experimental results are discussed in the next section.

## 5. Experimental Results

**Figure 10:**
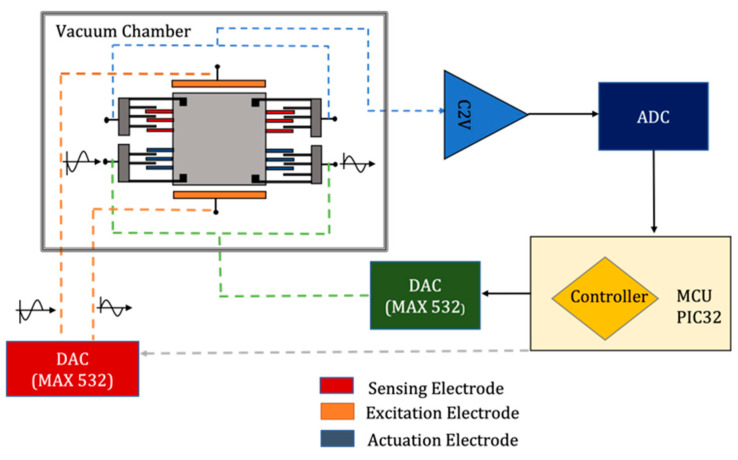
ISLX setup in the closed-loop configuration.

**Figure 11:**
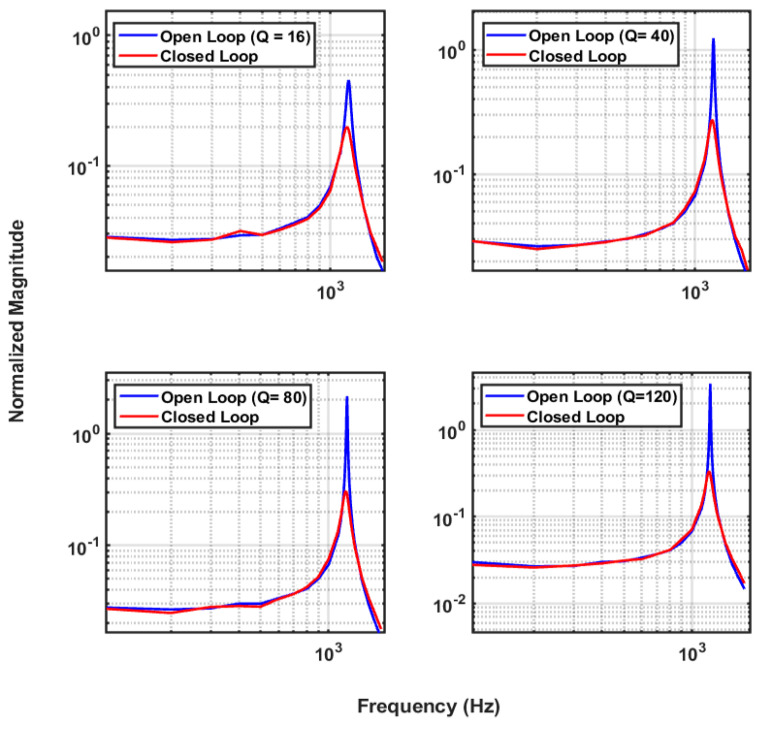
Frequency response of the ISL accelerometer in open-loop and closed-loop systems for excitation signals ranging from 50 to 1800 Hz.

To evaluate the performance of the closed-loop system, the setup illustrated in Figure 10 was used. Input excitation was applied through electrostatic forces generated by the top and bottom electrodes using two differential outputs from a DAC in form of a biased swept sine wave. One pair of sense electrodes was used for sensing and the other for actuation (bottom configuration in Figure 8). The sensor was placed inside a vacuum chamber. The output of the capacitance-to-voltage converter was digitized and transmitted to a computer for storage or display. The same digital output was then fed to the controller that was designed in the microcontroller. Feedback signals were then applied to the actuation electrodes through a separate pair of DAC outputs. The results shown here are for an input signal of 50 mV DC + 200 mV AC to simulate a 1 mg excitation acceleration. Test results for various vacuum levels ranging from 80 m Torr to 16 Torr were collected and are demonstrated in Figure 11. As much as 85% reduction in system response at resonance was recorded for a Q of 120.

**Figure 12:**
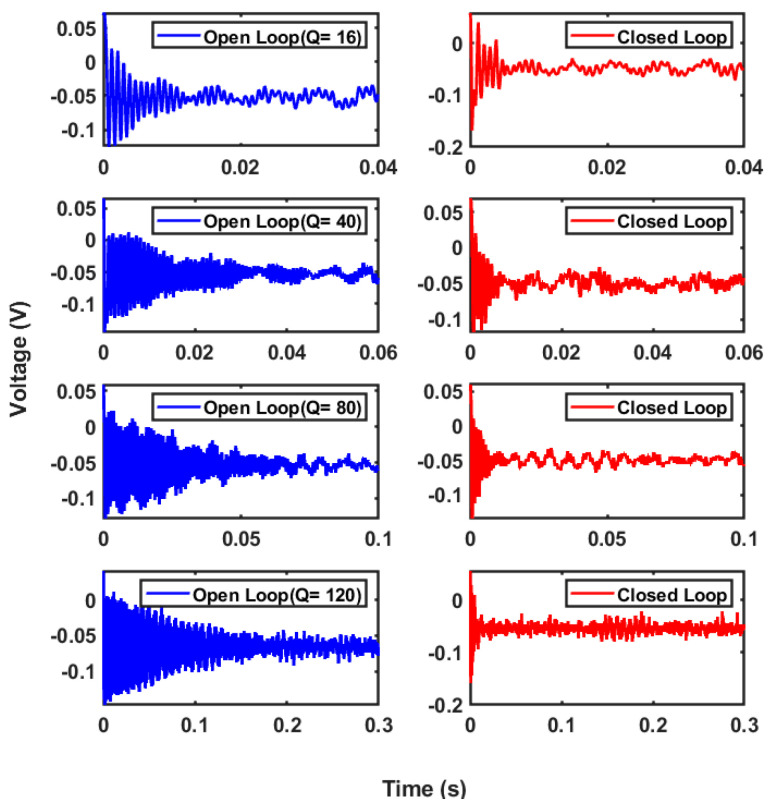
Time response of the sensor in the open-loop and closed-loop configuration for 1 mg of external excitation.

The same setup was used to study the step response of the system by applying a constant 7 V DC voltage for a short period. Figure 12 shows the time response result for both open- and closed-loop configurations for the same quality factor. The data clearly show that the system’s ringing increased as the open-loop system’s quality factor increased. However, a reduced oscillation was observed in the closed-loop system with the same quality factor as that of the open-loop system. The reduction in the output oscillation and settling time was estimated at around 85% in the closed-loop configuration compared to the open-loop configuration. Table 3 compares the performances of open and closed loop systems.

## 6. Conclusions

This paper presents a closed-loop control architecture of a highly underdamped ultra-sensitive accelerometer for suppressing large displacement. In order to produce feedback forces that could compensate for the large interfering accelerations around resonance, the accelerometer utilized two sets of electrodes that could be reconfigured on-the-fly to switch between high-sensitivity (open-loop) and high-resiliency (closed-loop) modes. The electrostatic damping technique is implemented to decrease the quality factor of the system at resonance artificially. A bandpass-based derivative controller is proposed and implemented to achieve the artificially damping technique. The results showed a good performance for suppressing large displacements due to external acceleration. Thus, the system showed less settling time and improved transient response.

## Figures and Tables

**Table 1:** Summary of high-Q accelerometers.

Author	Controller Type	Interface	Quality Factor (Q)	Noise Floor	Bandwidth, FS
Najafi [9]	2nd order ∑ΔM	Digital	16	1.5 $\mu g/\sqrt{\mathrm{Hz}}$	1 kHz, ±1.35 g
Aaltonen [18]	PID controller	Analog	30	–	300 Hz
Almut [10]	MASH	Digital	10	47 $\mu g/\sqrt{\mathrm{Hz}}$	1 kHz, ±1.5 g
Sarraf [16]	Sliding mode	Digital	9	3.3 $\mu g/\sqrt{\mathrm{Hz}}$	4.9 KHz
Yücetaş [13,14,15]	PD controller	Analog	>700	2 $\mu g/\sqrt{\mathrm{Hz}}$	200 Hz, ±1.5 g
Terzioğlu [12]	PI controller	Analog	3	~10 $\mu g/\sqrt{\mathrm{Hz}}$	200 Hz, ±3.5 g
Xu [11]	4th order ∑ΔM	Digital	40	200 $\mathrm{ng}/\sqrt{\mathrm{Hz}}$	300 Hz, ±1.2
Lavinia [17]	D controller	Digital	2000	0.62 $\mu g/\sqrt{\mathrm{Hz}}$	4.1 kHz
Chen [19]	PID + 5th order ∑ΔM	Hybrid (analog PID + digital EM)	200	1 $\mu g/\sqrt{\mathrm{Hz}}$	1 kHz
This work	Negative-derivative	Hybrid (analog interface + digital controller)	120	~50 $\mathrm{ng}/\sqrt{\mathrm{Hz}}$ (mech.) ~10 $\mu g/\sqrt{\mathrm{Hz}}$ (elec.)	1.2 kHz, ±3 g

**Table 2:** Physical characteristics of the ISLX.

Parameters	Values
Proof mass (mg)	8
Thickness (μm)	100
Dimension (length × width, ${\mu m}^{2}$)	6000 × 6000
Natural frequency (simulation, Hz)	1330
Natural frequency (experimental, Hz)	1280
Stiffness coefficient (N/m)	450
Overlap area per sensing finger, A $\left({\mu m}^{2}\right)$	720 × 100
Number of sensing electrodes per comb, N	69
Number of differential high-sensitivity comb pairs	2
Sense electrode gap, $d_{0}\;\left(\mu m\right)$	2
Overlap area per actuation electrode, $A_{a}$ $\left({\mu m}^{2}\right)$	6000 × 100
Actuation electrode gap, $d_{a}\;\left(\mu m\right)$	4.3
Pull-in voltage for high-sensitivity electrodes (V)	26
Pull-in voltage for low-sensitivity electrodes (V)	76

**Table 3:** Summary of the quantitative comparison between open-loop and closed-loop systems.

Open Loop		Closed Loop		% Reduction
Q	Settling time, $T_{s}\;\left(\mathrm{ms}\right)$	Q	Settling time, $T_{s}\;\left(\mathrm{ms}\right)$	Q
16	13.3	6.5	5.3	60
40	33.5	9	7.5	78
80	66.7	11.5	9.58	86
120	125	18	12	85

## Data Availability

Measurement data are available from the corresponding author upon request.
